# Breaking down barriers: A qualitative study of demand- and supply-side barriers to depression care in Nepal

**DOI:** 10.1017/gmh.2026.10236

**Published:** 2026-05-28

**Authors:** Nagendra P. Luitel, Poonam Sainju, Bishnu Lamichhane, Rajen Khadgi, Kamal Gautam

**Affiliations:** Research Department, Transcultural Psychosocial Organization (TPO) Nepal, Baluwatar, Kathmandu, Nepal

**Keywords:** Depression care, barriers, demand-side, supply-side, Nepal

## Abstract

Despite global initiatives like WHO’s mhGAP, mental health treatment gaps remain substantial, especially in LMICs. Barriers are often presented collectively, with an emphasis on supply-side solutions, while demand-side factors are frequently overlooked. Distinguishing these barriers and implementing tailored strategies is critical for improving access and utilization. This study explores stakeholders’ perceptions and experiences of demand- and supply-side barriers to mental healthcare in Nepal.

Qualitative interviews were conducted with 65 community stakeholders, including people with lived experience, using vignettes and the McGill Illness Narrative Interview (MINI) guide. Data were analyzed thematically in NVivo and interpreted through Levesque et al.’s access framework, which examines health system characteristics and individual capabilities.

Demand-side barriers included spiritual attributions of mental illness, stigma, low perceived need, financial hardship, lack of family support and limited awareness of conditions and available services. These factors hindered recognition, help-seeking, affordability and engagement. Supply-side barriers involved frequent staff transfers, inadequate training, lack of privacy, poor infrastructure and irregular psychotropic medicine supply, affecting service acceptability, availability and appropriateness.

Access to mental healthcare in Nepal is shaped by interconnected demand- and supply-side barriers. Addressing these requires culturally sensitive stigma-reduction, mental health literacy programs, workforce stabilization, reliable medication supply, privacy-friendly facilities and financial protection.

## Graphical abstract

**Figure:**
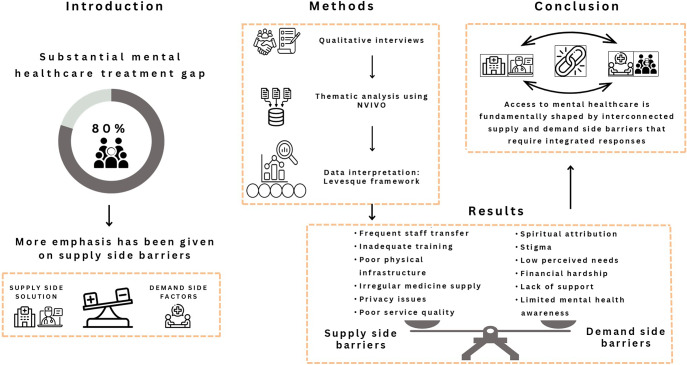

## Impact statement

This study explored the reasons why people with depression in Nepal struggle to access mental healthcare, despite efforts to integrate mental health services into the existing healthcare system. By examining barriers related to service availability and individual willingness to seek help, the study established how systemic issues within the healthcare system intersect with community beliefs, economic challenges and geographical constraints. The findings underscore that accessing mental health services in Nepal remains challenging due to various factors, including limited resources, inadequate staffing, inconsistent medication availability, as well as stigma, cultural beliefs and practices and a lack of awareness regarding where and when to seek support. Many individuals experienced barriers such as long travel distances, financial burdens and uncertainty about service availability in the nearby health facilities. Additionally, healthcare providers face obstacles like insufficient infrastructure, heavy workloads and limited support for delivering mental healthcare. The study contributes to the global mental health discourse by emphasizing that simply expanding services is insufficient to bridge the treatment gap. Effective mental healthcare requires strategies that bolster healthcare systems while addressing social, cultural and economic influences on help-seeking behavior. It highlights the importance of coordinated approaches involving community engagement, stigma reduction, culturally sensitive communication, improved supply chains and enhanced workforce capacity. By identifying these interconnected barriers and opportunities, the study offers practical insights to guide more effective and contextually relevant mental health policies and programs in Nepal and similar settings.

## Introduction

Despite global efforts to integrate mental health services into primary healthcare (PHC) systems, the treatment gap for mental health remains substantial, particularly in low- and middle-income countries (LMICs) (Patel et al., 2018; Petersen et al., 2019; Raviola et al., 2019). The World Health Organization (WHO) launched the Mental Health Gap Action Programme (mhGAP) Intervention Guide in 2010 to help with integration into primary care (WHO, 2010). While mhGAP has been implemented in over 100 countries (WHO, 2016), many individuals still do not receive treatment for common mental disorders like anxiety and mood disorders (Graham et al., 2017; Evans-Lacko et al., 2018). Patients with mood disorders often wait between 1 and 14 years for care, while those with anxiety disorders wait 3 to 30 years (Wang et al., 2007). These delays can increase health risks, reduce medication effectiveness and lower treatment success rates (Altamura et al., 2005, 2008; de Diego-Adeliño et al., 2010; Penninx et al., 2011; Bukh et al., 2013).

Access to healthcare is a multifaceted concept that involves individuals’ ability to obtain and utilize health services effectively (Gulliford et al., 2002). Penchansky and Thomas define access as the alignment between patients and the health system, encompassing five dimensions: availability, accessibility, accommodation, affordability and acceptability (Penchansky and Thomas, 1981). Andersen emphasizes actual service use and factors at individual, societal and system levels (Andersen, 1995). Levesque, Harris and Russell expand on these models, defining access as a dynamic process shaped by health system characteristics and individual abilities (Levesque et al., 2013). Their framework identifies dimensions such as approachability, acceptability, availability, accommodation, affordability and appropriateness paired with individuals’ abilities to perceive, seek, reach, pay and engage.

Global initiatives often focus on addressing supply-side barriers, such as increasing service availability and workforce capacity through task-sharing approaches (Endale et al., 2020; Keynejad et al., 2021; Dumke et al., 2024). However, research suggests that solely expanding services may not effectively reduce the treatment gap (Evans-Lacko et al., 2018; Fekadu et al., 2022). Barriers to mental healthcare are often oversimplified by combining demand- and supply-side factors. To effectively tackle this issue, it is crucial to differentiate between demand-side barriers and supply-side challenges and develop targeted strategies for each. For instance, public education and anti-stigma campaigns can increase willingness to seek care, while culturally sensitive interventions can enhance service delivery. Without this dual approach, investments in infrastructure and workforce may not lead to improved access and utilization, perpetuating global mental healthcare disparities (Troup et al., 2021; Bilican et al., 2025). This study aims to explore multi-stakeholder perceptions and experiences regarding barriers to accessing mental health services in Nepal. The results are analyzed using Levesque et al.’s access framework (Levesque et al., 2013), which considers both individuals’ ability to seek care and health system-level barriers.

## Methods

### Setting

Nepal is a low- and middle-income country in South Asia with a population of approximately 29.1 million and an annual growth rate of 0.92% (National Statistics Office, 2023). It is diverse in terms of caste/ethnicity and languages, with 142 caste ethnicities and 124 languages (National Statistics Office, 2023). The country adopted a federal governance system in 2015, establishing 7 provinces, 77 districts and 753 local units. This study was conducted in three districts, Jhapa, Chitwan and Kailali, selected for their diverse populations in terms of caste/ethnicity, language and healthcare delivery contexts (Lynn et al., 2008). Nepal’s healthcare system comprises public, private and non-governmental sectors, with specialized mental health services remaining scarce (Luitel et al., 2015; Mahat et al., 2018; Rai et al., 2021). Local governments deliver primary healthcare services through PHC facilities including, health posts, primary healthcare centers and hospitals. Community-based mental health services have been introduced using a task-sharing approach in collaboration with NGOs, and PHC workers in these districts have received WHO mhGAP training (Paudel et al., 2025).

### Study design

We conducted in-depth individual interviews (IDIs), a qualitative method ideal for exploring personal experiences and perspectives. The interviews were semi-structured with tailored guidelines for different participant groups. Community stakeholders such as teachers, traditional healers, female community health volunteers (FCHVs) and political leaders were presented with a narrative vignette depicting a person with depression to gather their views on help-seeking practices and perceived barriers. For individuals undergoing treatment for depression or anxiety, the interviews centered on their personal experiences and pathways to care.

### Participants and recruitment

The study was conducted with people receiving treatment for depression or anxiety (24), their family members (9), PHC providers (10), traditional healers (12), FCHVs, teachers and political leaders (10). Purposive sampling was used to ensure diversity in roles and experiences. Inclusion criteria were: (i) relevant role (patient, caregiver or provider), (ii) ability to communicate in Nepali, and (iii) willingness to share experiences. PHC workers and FCHVs were selected for their role in mental healthcare, while traditional healers were included to capture culturally embedded practices.

### Interview guides

Separate guides were developed for each participant group. Guides for individuals and families were adapted from the McGill Illness Narrative Interview (MINI), a semi-structured protocol for exploring illness experiences (Groleau et al., 2006; Craig et al., 2010). A vignette-based approach was used for community members to explore perceptions of depression, help-seeking pathways and available resources (Subba et al., 2017). PHC workers were asked about diagnostic practices, treatment approaches and interactions with patients.

### Interview process

Two trained researchers conducted all interviews in private settings chosen by participants to ensure comfort and confidentiality. Prior to interviews, participants received detailed information about study objectives, benefits and risks, and provided written informed consent. In adherence to the principle of non-maleficence, participants undergoing treatment for depression or anxiety were recruited through PHC workers or mental health professionals to prevent unnecessary disclosure or distress. Interviews were conducted either at participants’ homes or in a private clinical setting based on their preference to ensure their comfort and confidentiality. PHC workers were interviewed in outpatient departments during working hours. Family members were interviewed privately at home. Traditional healers were interviewed at their residences or service locations, and other community stakeholders at their workplaces. Interviewers were trained to handle discussions sensitively, and participants were informed of their right to pause, skip questions or withdraw at any point without impacting their care. Strict measures were in place to maintain privacy and confidentiality throughout the study. Participants experiencing psychological distress during the interview were offered free psychosocial support from trained counselors in their locality. Participants disclosing suicidal ideation were immediately connected with psychosocial support services. The management of suicidal ideation and other risks followed the Adverse Event Reporting and Management protocol (Singh et al., 2025). Data collection occurred between January and July 2023.

### Data management and analysis

Interviews were audio-recorded and transcribed immediately after each session. Professional translators translated the Nepali transcripts into English, and the first author reviewed translations for accuracy. Two researchers independently coded transcripts using NVivo 20 (QSR International). A hybrid inductive–deductive approach guided coding, informed by study objectives and emergent themes (Creswell and Poth, 2018). Thematic analysis was structured using the access framework by Levesque and colleagues (Levesque et al., 2013), which conceptualizes access as the interaction between health system characteristics (approachability, acceptability, availability/accommodation, affordability, appropriateness) and individual abilities (perceive, seek, reach, pay, engage). Certain findings were relevant to both demand- and supply-side barriers, as well as to different domains within either the demand or supply barriers. In these cases, the assignment of specific findings to multiple domains was decided through collaborative discussion and agreement between the first author and the research team.

## Results

Table 1 presents the socio-demographic characteristics of the study participants. Of the participants, 37% were undergoing treatment for depression or anxiety, 18.5% were traditional healers and 15% were PHC workers. A majority of the participants were female (52%), aged between 25 and 60 years (86%), married (86%) and had completed up to a secondary level of education (41.5%). The largest percentage of participants belonged to the Brahmin/Chhetri caste (52%), followed by Janajati (26%). The majority of participants were identified as Hindu. Among the participants, 40% were from Jhapa, 32% from Kailali and 28% from Chitwan.

**Table 1.** Socio-demographic characteristics of the participants Table 1. long description.

	Kailali *N* (%)	Chitwan *N* (%)	Jhapa *N* (%)	Total *N* (%)
**Participants type**				
Individuals receiving care for depression or anxiety	7 (33.33)	4 (22.22)	13 (50.00)	24 (36.92)
Caregiver	3 (14.29)	4 (22.22)	2 (7.69)	9 (13.85)
Traditional healer	2 (9.52)	3 (16.67)	7(26.92)	12 (18.46)
Primary healthcare worker	6 (28.57)	2 (11.11)	2 (7.69)	10 (15.38)
Other community stakeholder (teachers, FCHVs, political leaders)	3 (14.29)	5 (27.78)	2 (7.69)	10 (15.38)
**Sex**				
Male	12 (57.14)	7 (38.89)	15 (57.69)	34 (52.31)
Female	9 (42.86)	11 (61.11)	11 (42.31)	31 (47.69)
**Age**				
Below 25 years	1 (4.76)	1(5.56)	1 (3.85)	3 (4.62)
25–60 years	19 (90.48)	15 (83.33)	22 (84.62)	56 (86.15)
Above 60 years	1 (4.76)	2(11.11)	3 (11.54)	6 (9.23)
**Marital status**				
Married	15 (71.43)	18 (100.00)	23 (88.46)	56 (86.15)
Unmarried	5 (23.81)	0 (0.00)	0 (0.00)	5 (7.69)
Widow/widower	1 (4.76)	0 (0.00)	3 (11.54)	4 (6.15)
**Education**				
Illiterate	1 (4.76)	0 (0.00)	1 (3.85)	2 (3.08)
Literate only	2 (9.52)	3 (16.67)	3 (11.54)	8 (12.31)
Upto secondary level	6 (28.57)	7(38.89)	14 (53.85)	27 (41.54)
Intermediate and above	12 (57.14)	8 (44.44)	8 (30.77)	28 (43.08)
**Ethnicity**				
Brahmin/Chhetri	6 (28.57)	14 (77.78)	14 (53.85)	34 (52.21)
Janajati	8 (38.10	2(11.11)	7 (26.92)	17 (26.15)
Dalit	7(33.33)	2(11.11)	3 (11.54)	12 (18.46)
Madhesi/Meche	0 (0.00)	0 (0.00)	2 (7.69)	2 (3.08)
**Religion**				
Hindu	17 (80.95)	18 (100.00)	25 (96.15)	60 (92.31)
Christian	4 (19.05)	0 (0.00)	0 (0.00)	4 (6.16)
Prakritik (Limbu)	0 (0.00)	0 (0.00)	1 (3.85)	1 (1.54)
Total	21 (100.00)	18 (100.00)	26 (100.00)	65 (100.00)

**Figure 1.**
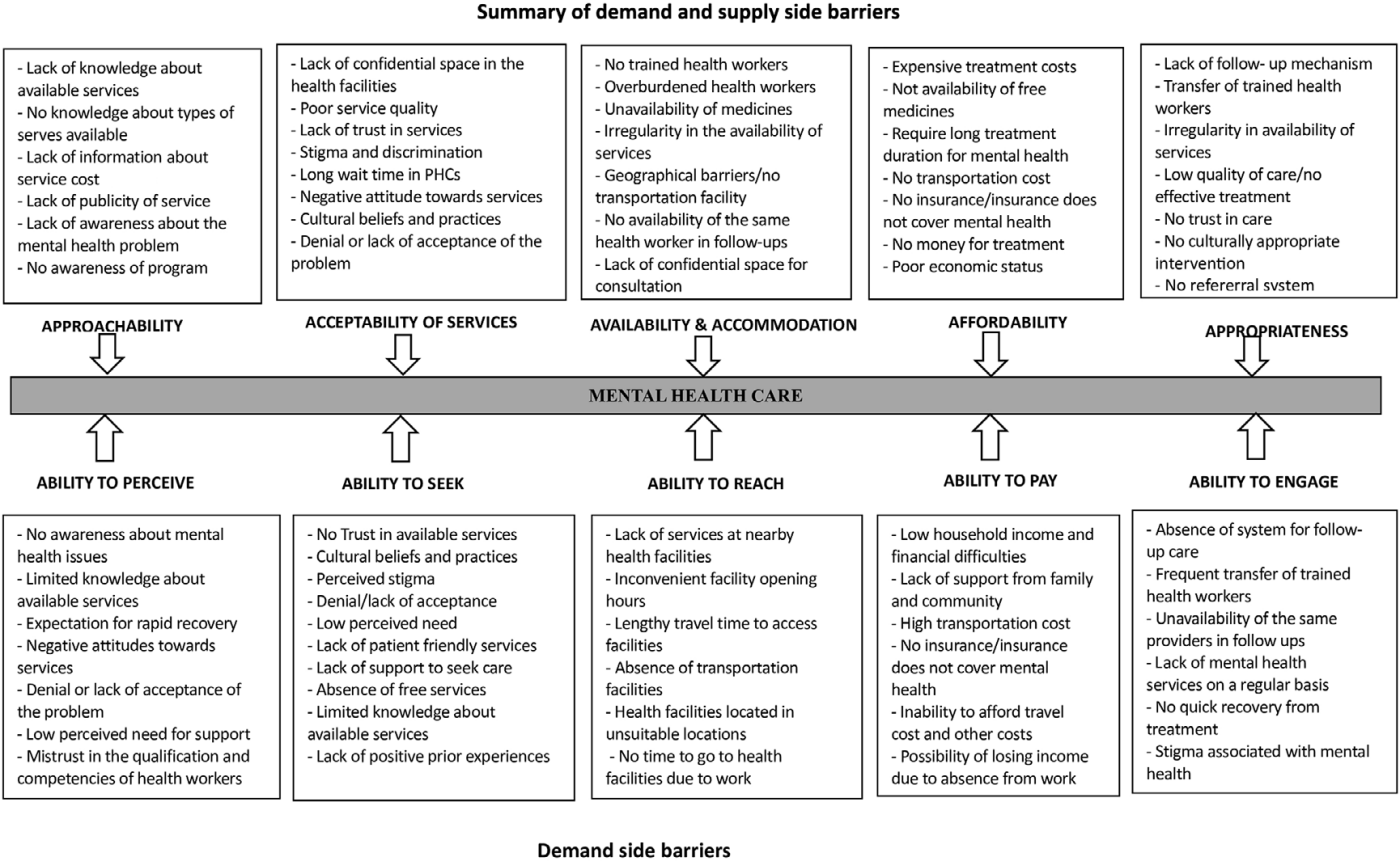
Summary of the demand- and supply side barriers. Figure 1. long description.

The barriers reported in the study are summarized in Figure 1 using the framework developed by Levesque and colleagues (Levesque et al., 2013). This framework comprises five supply-side barriers: approachability, acceptability of problems and services, availability and accommodation, affordability and appropriateness. It also includes five corresponding demand-side barriers related to perception, seeking, access, payment and engagement.

## Supply-side barriers

Participants identified a range of supply-side barriers across approachability, acceptability, availability and accommodation, affordability and appropriateness that collectively hindered timely and continuous mental healthcare. These barriers often interacted: limited visibility and stigma reduced initial engagement; organizational and logistical constraints disrupted continuity; financial barriers undermined treatment persistence; and gaps in provider capacity, referral systems and follow-up compromised care quality.

### Approachability of services

Approachability refers to how easily services can be identified and their benefits recognized. It was constrained by limited outreach and low visibility of mental health services relative to physical health programs. FCHVs reported that mental health remained a low-priority topic, reducing community awareness and proactive help-seeking. In such contexts, many community members turned to FCHVs rather than health-post staff.

The staff at the health post keep changing and come from different places. Honestly, people trust us more than the health post staff. KPBL_11

Lack of family knowledge also emerged as a major barrier, often delaying recognition of symptoms and timely access to care. One woman from Chitwan described feeling unsupported and unsure where to seek help.

If someone had informed me, I would have sought treatment earlier. No one in my family understands mental illness or guided me. PABL_11

Education level further influenced help-seeking. A health worker from Jhapa noted that individuals with more education were more likely to recognize problems and seek treatment promptly.

Educated individuals seek treatment and start medication faster. Uneducated individuals often delay. Mental illness affects both groups differently. HWBL_22

### Acceptability of problems and services

Acceptability, shaped by socio-cultural norms, stigma and confidentiality concerns, was a prominent barrier. Participants described how fear of recognition and disclosure discouraged facility attendance.

When someone goes to the health post, all the staff know their condition. People worry they might tell others, so they hesitate to go. KPBL_11

PHC workers highlighted that crowded out-patient departments and the lack of private rooms hindered trust-building and open communication.

If patients don’t talk openly, it’s difficult to help them. We try to earn trust, but in OPD they rarely share their history immediately. With many patients waiting, we rush, and they avoid returning. HWBL_03

Traditional beliefs attributing distress to supernatural causes further discouraged biomedical care.

I doubt medicine will help. I think my condition may be due to ghosts, curses, or black magic, so I only consider medicine in extreme cases. PAPS_01

Preference for traditional healers reinforced delays.

I also believed in superstitions, thinking my nighttime fears caused my illness. These beliefs prevent people from getting proper treatment. People need to trust science over superstition. PABL_02

### Availability and accommodation

Availability and accommodation reflecting organization, staffing, infrastructure and logistics were widely cited constraints. Participants described stock-outs of free medications, frequent staff transfers, inadequately equipped facilities, crowded outpatient departments, limited confidential spaces and geographical barriers. Stock-outs particularly frustrated patients expecting free medicines at public facilities.

Patients go where they can get medicines. Just yesterday, one woman said, “we go to government facilities but don’t get medicines, so we end up buying them privately”. HWBL_03

Human resources were insufficient for demand, and frequent rotation of trained providers disrupted continuity and trust.

My colleagues aren’t trained in mental health. They need my advice even after seeing patients because I’m the only trained prescriber. HWPS_11

Lack of private consultation rooms limited open disclosure.

They feel hesitant. They may meet neighbors at the facility and don’t feel safe. There’s no separate department or room, so they can’t share everything. I’ve seen this often. HWBL_04

Overcrowding and overworked staff led to rushed consultations, discouraging return visits.

The doctor had many patients and came only once every 1–2 months. There were over 200 patients; we were seen at midnight. The consultation was rushed—just a few questions, no proper counseling. PABL_24

Limited awareness of mental health services in primary care led some individuals to seek treatment outside Nepal.

I didn’t know mental health services were available in Nepal. My friend and I went to India because he also didn’t know he could get treatment from primary health workers. PAPS_01

### Affordability of services

Affordability constraints, including medicine costs, travel expenses and inconsistent insurance coverage – compromised treatment initiation and adherence. Health workers described how the lack of free psychotropic medicines in public facilities forced patients to rely on out-of-pocket purchases over long periods, often leading to treatment discontinuation.

He [patient] has to take medicine regularly. He said he could buy it once or twice, but buying it every time is difficult. HWPS_11

Traditional healers were viewed as more affordable, influencing care-seeking pathways.

People avoid doctors thinking it will cost a lot. Going to a traditional healer cost much less… Poor people cannot afford a doctor. KPBL_21

Travel costs further restricted follow-up and continuity.

I was advised to go to Kathmandu or Nepalgunj for possible neurological illness, but I couldn’t afford the travel. Kailali has no neurological doctors, so finances limited my access to specialized care. PAPS_08

Insurance gaps added to these affordability barriers, with inconsistent availability of mental health services in government hospitals and many private hospitals not accepting insurance.

Most government hospitals accept insurance, but many private ones, including the hospital I visited, don’t. I’m also not aware which government hospitals provide mental health services. PABL_24

Supply-side financing and procurement issues further contributed to medicine shortages.

There may be a dozen free mental health medicines, but they don’t reach our center. Local governments now manage purchases, but they haven’t consulted us. HWBL_22

### Appropriateness of services

Appropriateness covering fit, quality, continuity and timeliness was weakened by perceived gaps in provider’s competence, inconsistent service delivery and management limitations. Some patients questioned professionalism and clinical quality.

When I shared my symptoms, he kept asking questions, writing, and calling his superior to ask what to do. Sometimes he looked at his phone. I didn’t feel confident; he seemed too young and inexperienced. PABL_42

PHC workers described how few trained service providers led to heavy workloads, delays and limited follow-up.

The lack of proper management, few trained staff, and inadequate lab facilities may be the reason. People also don’t come for follow-up on their own. HWPS_11

Frequent staff transfers disrupted continuity, leaving gaps when trained personnel moved away.

We sent one staff for training in Dadeldhura, but he was transferred soon after. If a trained worker stays, services improve, but once they leave, quality drops. HWBL_03

Medication supply issues such as delayed delivery, limited stock and short-expiry medicines further undermined care.

When we ask the municipality about mental health medicines, they say the province purchases them. But the medicines often arrive with only six months’ expiry left… and we must discard them after that. HWBL_11

Participants emphasized the lack of structured follow-up and referral systems, leading to discontinuities, mistrust and severe outcomes.

There was a postpartum depression case. I suspected a mental health issue and informed the nurses. We provided counseling, but while trying to link her to a doctor, she committed suicide. This happened because we lack a proper referral mechanism. We can diagnose cases, but without a system to connect them to treatment, we face serious challenges. HWBL_22

## Demand side barriers

Demand-side barriers encompass five dimensions: perception, seeking, access, affordability and engagement, hindering the consistent utilization of mental health services. These barriers are rooted in socio-cultural norms, financial constraints and systemic shortcomings, leading to delays, gaps and distrust in the healthcare system.

### Ability to perceive

The ability to perceive refers to individuals’ capacity to recognize mental health needs and the importance of care. Cultural beliefs, stigma and low perceived need were major obstacles. A strong reliance on traditional practices and superstitions often delayed or prevented professional care:

I, too, have faith in superstitions. This dependence on superstitions can prevent individuals from seeking appropriate medical treatment. These beliefs have been handed down through generations and greatly influence our healthcare choices. PABL_02

Traditional healers also reported that many individuals consult them before visiting hospitals.

In the past, it was common to seek the help of traditional healers (Dhami-jhakri) and have faith in their healing abilities. This tradition continues today, as people still visit traditional healers and perform rituals before seeking medical treatment at hospitals. KPBL_41

Stigma and discrimination further shaped perceptions, discouraging help-seeking. Patients feared being labeled “mad” and concealed their illness.

I lied to the villagers, telling them I was taking medicine for headaches to avoid negative judgment. Nobody wants to be labeled as mad. PABL_42

Low perceived need was another barrier. Many patients delayed treatments until symptoms became severe.

Mostly I go to the hospital only when it becomes very difficult, otherwise I go when my medicines are finished. If the pain is tolerable, then I do not go to the hospital. PABL_11

Denial of illness was repeatedly cited as a major barrier, contributing to delayed treatment and dropout cases.

First of all, none of the mental health patients ever feel that they have a mental health illness or that they have a mental health problem. They never accept this fact. HWBL_03

Lack of awareness about mental health was pervasive. Patients often misunderstood symptoms, assuming physical causes such as heart problems and sought treatment accordingly.

If someone has depression, they are usually unaware of it. They start visiting doctors thinking they have heart issues. HWBL_21

Patients themselves echoed this struggle of a lack of awareness about mental health issues.

I didn’t understand what was happening to me or what I should do. I never imagined I would go through this. PABL_11

### Ability to seek

The ability to seek reflects individuals’ capacity to identify and choose appropriate services. Participants mentioned barriers such as frequent staff turnover, lack of private spaces, poor facility conditions, distrust in services and stigma. The absence of privacy made individuals hesitant to disclose information.

They feel reluctant… There is no secure and separate area or room; they are unable to share all their information. HWBL_04

Patients with depression found crowded and noisy facilities stressful.

They couldn’t manage to go to the hospital alone… It would only increase their stress if they went alone. The hospital is noisy and crowded, adding to their stress. CGPS_12

Participants also highlighted stigma and misconceptions, which hindered help-seeking.

In rural areas, many conceal mental health issues out of fear of being labeled as ’crazy’ or ’half-brained.’ They seek help from traditional healers for problems like insomnia, attributing it to divine displeasure. KPPS_01

### Ability to reach

Ability to reach refers to individuals’ capacity to physically access services. Geographic barriers – such as long distances, poor road conditions and limited transportation were among the most frequently cited challenges. Health workers noted that long travel distances discourage patients from visiting health facilities. Unpaved roads and lengthy travel times make regular visits inconvenient.

We still have challenges, as the roads are not all paved, and the patients have to travel on foot till here, which takes a long time. HWBL_05

Several participants reported that traveling to India was easier than reaching Kathmandu.

Lucknow is near… it takes only five hours. To reach Kathmandu from here, it takes 18–22 hours. Distance is also the reason they go to India… and it is easily accessible. HWBL_06

Patients from Kailali noted that treatment in India is considered more accessible, affordable and technologically advanced.

India is close to here, and when comparing, it’s also cheaper than Kathmandu. Traveling back and forth is easier. PABL_05

### Ability to pay

Financial constraints were a major barrier to maintaining treatment. The lack of free medicines, high travel costs and insufficient family support often resulted in treatment discontinuation. Family financial difficulties compounded these issues.

When I went for a check-up for my depression, my husband and mother-in-law refused to give me money… I couldn’t go to the follow-up because I didn’t have the money and I also had to discontinue the medication. PAPS_04

Even when families were willing to provide support, secrecy surrounding illness made it difficult for patients to ask for help.

It should be free. Even if I have to pay for the medicine, I ought to take it. So, I lie to my son, I lie to my daughter-in-law and tell them, ‘I have to go somewhere today. Please give me money for the bus fare.’ And from that money, I buy my medicine. PABL_12

Traditional healers observed that financial hardship drives patients toward more affordable traditional practices.

Patients don’t go to doctors thinking it will cost a lot. Visiting a traditional healer (dhami) is cheap. If the healer can cure them, why face trouble at the hospital? Poor people can’t afford doctors; that’s why they go to healers. KPBL_21

### Ability to engage

Ability to engage refers to individuals’ motivation and capacity to participate in decision-making and remain committed to treatment. Major barriers included mistrust in services, financial constraints, geographical challenges and systemic issues. Participants frequently reported doubts about health workers’ competence, particularly when diagnoses were unclear or poorly communicated, leaving them uncertain about their medications.

When I first started taking the medicine… I came across a similar one used for depression. Then doubts came to my mind, like whether this medicine will harm me or whether they are concealing my real condition. PABL_02

Frequent transfers of PHC workers disrupted continuity of care and discouraged follow-up, compounded by limited trained staff and lack of refresher training.

Some health workers were transferred or promoted… Seeing different staff at each visit made the process less reliable and discouraged treatment. HWBL_11

Financial barriers also restricted engagement. Many patients sought government services only after depleting resources at private hospitals. The absence of free medicines further diminished trust.

If free medicines are not available, they lose trust in the health system and are discouraged from seeking further treatment. HWBL_05

Similarly, a traditional healer from Jhapa highlighted the role of financial hardship and cultural beliefs in disengagement.

To visit the doctor, you need a bit more money and many people don’t even have money. Now not much money is required for ritual blowing (jharfuk), so they trust it, have blind faith, and go there. So many accidents have occurred going to such places too. KPBL_44

Participants also highlighted compromised services and a lack of patient-friendly environments at health posts. Crowded, noisy facilities made patients anxious and discouraged visits.

Hospitals are noisy and crowded. It increases tension for patients. CGPS_12

## Discussion

The findings indicate that access to mental health services in Nepal is constrained by interlinked supply- and demand-side barriers. On the supply side, acceptability is limited by stigma, confidentiality concerns, traditional beliefs and spiritual explanatory models (Kohrt et al., 2005; Luitel et al., 2025) while privacy, dignity and social status strongly influence help-seeking behaviors (Brenman et al., 2014; Gurung et al., 2022). Affordability issues arise from the lack of free medications, out-of-pocket expenses and travel costs, due to under-funding and inconsistent medicine availability (Brenman et al., 2014; Paudel et al., 2025). Limited outreach and visibility of mental health services compared to physical health programs contribute to reduced approachability, leading to awareness gaps even among educated individuals (Brenman et al., 2014; Khanal et al., 2025). Availability and accommodation are hindered by stock-outs, staff transfers, high workloads, inadequate confidential spaces and geographic constraints (Khanal et al., 2025; Paudel et al., 2025). Perceived gaps in provider competence and unreliable medication supply compromise appropriateness and trust (Hynie et al., 2022). On the demand side, stigma and supernatural beliefs limit perceived need, while mistrust, distance, transportation costs, financial hardship and discontinuity of care impede service uptake and sustained engagement (Gurung et al., 2022; van den Broek et al., 2023).

Low detection of mental health problems by trained PHC workers is a global phenomenon (Fekadu et al., 2022; Kohrt et al., 2025), and Nepal is no exception to this global situation. Existing studies in Nepal have revealed that trained PHC workers were able to detect less than one in four people (Jordans et al., 2019; Kohrt et al., 2025), which is much higher than the rate reported in other countries (Fekadu et al., 2022). The lack of a confidential space for diagnosis, counseling and treatment, especially for mental healthcare services at local health facilities, has been identified as a major barrier that reduces access to mental healthcare services and increases dropout rates among service users (Luitel et al., 2020; Upadhaya et al., 2020). Our study also found that the lack of confidential space is a significant barrier preventing service users from seeking mental healthcare treatment, which ultimately impacts the quality and effectiveness of the services provided. The absence of separate and confidential consultation rooms leads to a loss of trust among service users regarding the confidentiality of their information, especially discouraging patients who are concerned about their privacy. This finding is consistent with other studies (Luitel et al., 2020; Devkota et al., 2021).

Another supply-side barrier highlighted in the study is the frequent transfer of trained PHC workers in local healthcare facilities. This practice not only disrupts the delivery of mental healthcare services but also contributes to high dropout rates and community mistrust (Upadhaya et al., 2020). The shortage of experienced PHC workers in facilities creates challenges in offering consistent mental health services (Jordans et al., 2019; Luitel et al., 2020; Upadhaya et al., 2020; Devkota et al., 2021). This issue has been extensively addressed in previous studies and reports on expanding mental health services (Luitel et al., 2020; Upadhaya et al., 2020). The resulting lack of trust between mental health service providers and users due to frequent staff transfers significantly impacts mental health service utilization in Nepal (Devkota et al., 2021).

Demand-side factors also significantly impact the utilization of mental healthcare services. The low perceived need for treatment among individuals seeking help has been consistently identified as a major barrier to accessing mental healthcare services in Nepal, leading to a significant treatment gap (Luitel et al., 2017). Factors such as mental health stigma, lack of awareness and the prioritization of other health issues over mental health contribute to this low perceived need for treatment among service users (Luitel et al., 2017, 2019; Rai et al., 2021), resulting in delayed or inadequate treatment seeking. Moreover, the perception of ineffective treatment among service users is another barrier to service utilization in Nepal. Studies have shown that the decision to seek mental health treatment and engage with it is closely linked to patients’ concerns about the effectiveness of mental health interventions (Luitel et al., 2017, 2020). This perception may be influenced by cultural beliefs about mental health and treatment efficacy, as well as past negative experiences, further discouraging individuals from accessing services (Luitel et al., 2020).

The findings of this study could have implications for improving access to mental health services in Nepal. Despite the Government of Nepal’s efforts to integrate mental health into the PHC system, our findings suggest that simply making services available does not automatically lead to increased utilization. This aligns with previous studies that have shown integration efforts facing systemic barriers beyond individual provider skills (Upadhaya et al., 2020). Persistent challenges on the supply-side, such as frequent staff turnover, shortages of mhGAP-trained personnel, inadequate supervision structures and insufficient private spaces in health facilities, continue to hinder effective access (Luitel et al., 2020). Addressing these issues requires strengthening workforce stability by integrating WHO mhGAP training into the pre-service education of future healthcare providers, providing in-service clinical supervision and refresher training and strengthening local governance to ensure the stable placement of trained PHC workers at the community level. Additionally, establishing supportive supervision mechanisms and upgrading infrastructure to include confidential consultation rooms are essential (Upadhaya et al., 2020). Second, ensuring a reliable supply chain of psychotropic medicines is crucial. Frequent stock-outs and short expiry periods disrupt treatment continuity and discourage ongoing care. The Nepal health facility survey has reported low psychotropic medicine readiness scores (around 30%), indicating weaknesses in procurement and distribution systems (Acharya et al., 2025). Third, financial reforms are necessary to reduce out-of-pocket expenses for medications, transportation and referrals. Many patients turn to traditional healers or discontinue biomedical treatment due to the high costs involved (Dhimal et al., 2022). Strengthening national health insurance programs by including psychotropic medicines and subsidizing travel expenses for specialist referrals could help alleviate the burden of catastrophic health expenditures. These priorities are in line with WHO recommendations and recent analyses of systemic constraints in Nepal (Paudel et al., 2025).

On the demand side, addressing stigma, cultural explanatory models of mental health problems and low levels of mental health literacy is equally critical. Stigma remains a dominant barrier, contributing to delayed care-seeking and high dropout rates (Upadhaya et al., 2020). Community-based programs, including anti-stigma activities delivered by trusted local actors such as FCHVs, teachers, religious leaders and community representatives, could encourage early identification and help-seeking behaviors. To overcome geographical barriers and challenges in follow-up care, telepsychiatry and structured teleconsultation using e-mhGAP tools (Kohrt et al., 2025) can extend specialist input to peripheral facilities and support continuity of care when trained staff are rotated (Shakya, 2021). Early pilots of mobile-based mhGAP tools in Nepal demonstrate feasibility, acceptability and the potential to improve diagnostic consistency; however, sustained investments in digital infrastructure and supervision fidelity remain essential (Luitel et al., 2023). Finally, aligning national mhGAP roll-out with the updated 2023 WHO guidelines (WHO, 2023) and Nepal’s evolving National Mental Health Strategy and Action Plan (MoHP, 2020) can help standardize care pathways and strengthen accountability across federal, provincial and municipal tiers.

Several limitations warrant consideration. First, although the study was conducted in three districts covering the eastern, central and western parts of the country, considering the diversity of Nepal in terms of geography, culture, caste/ethnicity and languages, the result of the study may not be representative of the entire country. Second, social desirability and fear of disclosure may have led to under-reporting of stigma or provider behaviors, particularly given confidentiality concerns that participants themselves described. Third, as the study was conducted in the districts where mental health services were available, the results could be different in the districts where those services are not available. Fourth, in this study, our focus was primary with depression; the challenges and barriers reported in this study could be different for other disorders, particularly for severe mental health problems.

Finally, while we used Levesque’s framework to synthesize the results, this framework does not explicitly capture multiple dimensions of help-seeking barriers, such as external and internal contextual factors, the intervention itself, its implementation and interactions between users and the intervention.

## Conclusion

This study reveals persistent and inter-related supply- and demand-side barriers that significantly hinder access to mental healthcare in Nepal. On the supply side, challenges include inconsistent staffing, frequent medication shortages, inadequate infrastructure and limited prioritization of mental health within the health system. Demand-side barriers are shaped by stigma, cultural misconceptions, financial hardship and geographic inaccessibility. Addressing these obstacles requires a coordinated, multi-sectoral/multi-level response: strengthening health system capacity through workforce development, reliable procurement and distribution of psychotropic medications and improved clinical infrastructure; coordination and collaboration with non-health sectors, alongside targeted community-level strategies such as culturally sensitive awareness campaigns, stigma reduction initiatives, financial protection measures and active community engagement. Closing Nepal’s mental health treatment gap demands a comprehensive, integrated approach that not only addresses structural limitations but also respects socio-cultural realities.

## Data Availability

Interested individuals can contact the principal investigator of this study to express their interest in collaboration and request access to the dataset analyzed here by emailing: luitelnp@gmail.com.

## Abbreviations

FCHV: Female Community Health Volunteer
HP: Health Post
mhGAP-IG: Mental Health Gap Action Programme Intervention Guide
NGO: Non-governmental Organization
OPD: Outpatient Department
PHC: Primary Healthcare
WHO: World Health Organization

## Table 1. Long description

The table is organized by row groups for participant type, sex, age, marital status, education, ethnicity, and religion. Each group contains subcategories listed in the first column. For each subcategory, counts and percentages are provided for Kailali, Chitwan, Jhapa, and the total. For participant type, individuals receiving care for depression or anxiety are 7 (33.33 percent) in Kailali, 4 (22.22 percent) in Chitwan, 13 (50.00 percent) in Jhapa, total 24 (36.92 percent). Caregivers are 3 (14.29 percent), 4 (22.22 percent), 2 (7.69 percent), total 9 (13.85 percent). Traditional healers are 2 (9.52 percent), 3 (16.67 percent), 7 (26.92 percent), total 12 (18.46 percent). Primary healthcare workers are 6 (28.57 percent), 2 (11.11 percent), 2 (7.69 percent), total 10 (15.38 percent). Other community stakeholders are 3 (14.29 percent), 5 (27.78 percent), 2 (7.69 percent), total 10 (15.38 percent). For sex, males are 12 (57.14 percent), 7 (38.89 percent), 15 (57.69 percent), total 34 (52.31 percent); females are 9 (42.86 percent), 11 (61.11 percent), 11 (42.31 percent), total 31 (47.69 percent). For age, below 25 years are 1 (4.76 percent), 1 (5.56 percent), 1 (3.85 percent), total 3 (4.62 percent); 25–60 years are 19 (90.48 percent), 15 (83.33 percent), 22 (84.62 percent), total 56 (86.15 percent); above 60 years are 1 (4.76 percent), 2 (11.11 percent), 3 (11.54 percent), total 6 (9.23 percent). For marital status, married are 15 (71.43 percent), 18 (100.00 percent), 23 (88.46 percent), total 56 (86.15 percent); unmarried are 5 (23.81 percent), 0 (0.00 percent), 0 (0.00 percent), total 5 (7.69 percent); widows are 1 (4.76 percent), 0 (0.00 percent), 3 (11.54 percent), total 9 (13.85 percent). For education, illiterate are 1 (4.76 percent), 0 (0.00 percent), 1 (3.85 percent), total 2 (3.08 percent); literate only are 2 (9.52 percent), 3 (16.67 percent), 3 (11.54 percent), total 8 (12.31 percent); up to secondary level are 6 (28.57 percent), 7 (38.89 percent), 14 (53.85 percent), total 27 (41.54 percent); intermediate and above are 12 (57.14 percent), 8 (44.44 percent), 8 (30.77 percent), total 28 (43.08 percent). For ethnicity, Brahmin/Chhetri are 6 (28.57 percent), 14 (77.78 percent), 14 (53.85 percent), total 34 (52.21 percent); Janajati are 8 (38.10 percent), 2 (11.11 percent), 7 (26.92 percent), total 17 (26.15 percent); Dalit are 7 (33.33 percent), 2 (11.11 percent), 3 (11.54 percent), total 12 (18.46 percent); Madhesi/Meche are 0 (0.00 percent), 0 (0.00 percent), 2 (7.69 percent), total 2 (3.08 percent). For religion, Hindu are 17 (80.95 percent), 18 (100.00 percent), 25 (96.15 percent), total 60 (92.31 percent); Christianity are 4 (19.05 percent), 0 (0.00 percent), 0 (0.00 percent), total 4 (6.16 percent); Prakritik (Limbu) are 0 (0.00 percent), 0 (0.00 percent), 1 (3.85 percent), total 1 (1.54 percent). The final row shows total participants: 21 (100.00 percent) in Kailali, 18 (100.00 percent) in Chitwan, 26 (100.00 percent) in Jhapa, total 65 (100.00 percent).

Navigate back to Table 1..

## Figure 1. Long description

From left to right, the flowchart is divided into five vertical columns: approachability, acceptability of services, availability and accommodation, affordability, and appropriateness. Each column contains two stacked boxes. The top box in each column lists supply side barriers, such as lack of knowledge about available services, lack of confidential space, no trained health workers, expensive treatment costs, and lack of follow-up mechanisms. The bottom box in each column lists demand side barriers, including no awareness about mental health issues, no trust in available services, lack of services at nearby facilities, low household income, and absence of follow-up care. Arrows connect each barrier type to a central horizontal bar labeled mental health care, which is itself divided into five segments corresponding to the five dimensions. Each segment is labeled with the associated ability: ability to perceive, ability to seek, ability to reach, ability to pay, and ability to engage. The diagram visually aligns supply and demand barriers with their respective dimensions of access to mental health care.

Navigate back to Figure 1..

## References

[r1] Acharya K, Singh D, Karki A, Cleary M and Thapa D (2025) Mental health service readiness in Nepal: Insights from the 2021 Nepal Health Facility Survey. PLOS Mental Health 2(7), e0000155. 10.1371/journal.pmen.0000155 41661939 PMC12798464

[r2] Altamura AC, Dell’osso B, D’Urso N, Russo M, Fumagalli S and Mundo E (2008) Duration of untreated illness as a predictor of treatment response and clinical course in generalized anxiety disorder. CNS Spectrums 13(5), 415–422. 10.1017/s1092852900016588. 18496479

[r3] Altamura AC, Santini A, Salvadori D and Mundo E (2005) Duration of untreated illness in panic disorder: A poor outcome risk factor? Neuropsychiatric Disease and Treatment 1(4), 345–347 18568114 PMC2424121

[r4] Andersen RM (1995) Revisiting the behavioral model and access to medical care: Does it matter? Journal of Health and Social Behavior 36(1), 1–10. 10.2307/2137284 7738325

[r5] Bilican S, Irfan M, Cox A, Salaets H, Sabbe M and Schoenmakers B (2025) Access to mental healthcare for refugees, asylum seekers and migrants: An umbrella review of barriers. BMJ Open 15(6), e096267. 10.1136/bmjopen-2024-096267 PMC12161349 40484433

[r6] Brenman NF, Luitel NP, Mall S and Jordans MJD (2014) Demand and access to mental health services: A qualitative formative study in Nepal. BMC International Health and Human Rights 14(1), 22. 10.1186/1472-698x-14-22 25084826 PMC4126616

[r7] Bukh JD, Bock C, Vinberg M and Kessing LV (2013) The effect of prolonged duration of untreated depression on antidepressant treatment outcome. Journal of Affective Disorders 145(1), 42–48. 10.1016/j.jad.2012.07.008 22854096

[r8] Craig SR, Chase L and Lama TN (2010) Taking the MINI to mustang, Nepal: Methodological and epistemological translations of an illness narrative interview tool. Anthropology & Medicine 17(1), 1–26. 10.1080/13648471003602566 20419514

[r9] Creswell JW and Poth CN (2018) Qualitative Inquiry and Research Design Choosing among Five Approaches, 4th Edition, SAGE Publications, Inc., Thousand Oaks.

[r10] de Diego-Adeliño J, Portella MJ, Puigdemont D, Pérez-Egea R, Alvarez E and Pérez V (2010) A short duration of untreated illness (DUI) improves response outcomes in first-depressive episodes. Journal of Affective Disorders 120(1–3), 221–225. 10.1016/j.jad.2009.03.012 19349077

[r11] Devkota G, Basnet P, Thapa B and Subedi M (2021) Factors affecting utilization of mental health services from primary health care (PHC) facilities of western hilly district of Nepal. PLoS One 16(4), e0250694. 10.1371/journal.pone.0250694 33930894 PMC8087454

[r12] Dhimal M, Dahal S, Adhikari K, Koirala P, Bista B, Luitel N, et al. (2022) A Nationwide prevalence of common mental disorders and suicidality in Nepal: Evidence from National Mental Health Survey, 2019-2020. Journal of Nepal Health Research Council 19(04), 740–747. 10.33314/jnhrc.v19i04.4017 35615831

[r13] Dumke L, Wilker S, Hecker T and Neuner F (2024) Barriers to accessing mental health care for refugees and asylum seekers in high-income countries: A scoping review of reviews mapping demand and supply-side factors onto a conceptual framework. Clinical Psychology Review 113, 102491. 10.1016/j.cpr.2024.102491 39213812

[r14] Endale T, Qureshi O, Ryan GK, Esponda GM, Verhey R, Eaton J, et al. (2020) Barriers and drivers to capacity-building in global mental health projects. International Journal of Mental Health Systems 14(1), 89. 10.1186/s13033-020-00420-4 33292389 PMC7712613

[r15] Evans-Lacko S, Aguilar-Gaxiola S, Al-Hamzawi A, Alonso J, Benjet C, Bruffaerts R, et al. (2018) Socio-economic variations in the mental health treatment gap for people with anxiety, mood, and substance use disorders: Results from the WHO world mental health (WMH) surveys. Psychological Medicine 48(9), 1560–1571. 10.1017/s0033291717003336 29173244 PMC6878971

[r16] Fekadu A, Demissie M, Birhane R, Medhin G, Bitew T, Hailemariam M, et al. (2022) Under detection of depression in primary care settings in low and middle-income countries: A systematic review and meta-analysis. Systematic Reviews 11(1), 21. 10.1186/s13643-022-01893-9 35123556 PMC8818168

[r17] Graham A, Hasking P, Brooker J, Clarke D and Meadows G (2017) Mental health service use among those with depression: An exploration using Andersen’s behavioral model of health service use. Journal of Affective Disorders 208, 170–176. 10.1016/j.jad.2016.08.074 27788380

[r18] Groleau D, Young A and Kirmayer LJ (2006) The McGill Illness Narrative Interview (MINI): An interview schedule to elicit meanings and modes of reasoning related to illness experience. Transcult Psychiatry 43(4), 671–691. 10.1177/1363461506070796 17166953

[r19] Gulliford M, Figueroa-Munoz J, Morgan M, Hughes D, Gibson B, Beech R and Hudson M (2002) What does ’access to health care’ mean? Journal of Health Services Research & Policy 7(3), 186–188. 10.1258/135581902760082517 12171751

[r20] Gurung D, Poudyal A, Wang YL, Neupane M, Bhattarai K, Wahid SS, et al. (2022) Stigma against mental health disorders in Nepal conceptualised with a ’what matters most’ framework: A scoping review. Epidemiology and Psychiatric Sciences 31, e11. 10.1017/s2045796021000809 35086602 PMC8851063

[r21] Hynie M, Jaimes A, Oda A, Rivest-Beauregard M, Perez Gonzalez L, Ives N, et al. (2022) Assessing virtual mental health access for refugees during the COVID-19 pandemic using the Levesque client-centered framework: What have we learned and how will we plan for the future? International Journal of Environmental Research and Public Health 19(9), 5001. 10.3390/ijerph19095001 35564397 PMC9103707

[r22] Jordans MJD, Luitel NP, Kohrt BA, Rathod SD, Garman EC, De Silva M, et al. (2019) Community-, facility-, and individual-level outcomes of a district mental healthcare plan in a low-resource setting in Nepal: A population-based evaluation. PLoS Medicine 16(2), e1002748. 10.1371/journal.pmed.1002748. 30763321 PMC6375569

[r23] Keynejad R, Spagnolo J and Thornicroft G (2021) WHO mental health gap action programme (mhGAP) intervention guide: Updated systematic review on evidence and impact. Evidence-Based Mental Health 24(3), 124–130. 10.1136/ebmental-2021-300254 33903119 PMC8311089

[r24] Khanal G, Selvamani Y and Sapkota P (2025) Exploring barriers and facilitators of mental health care in Sudurpaschim Province, Nepal: A socioecological qualitative study of patients with depression and anxiety and health care professionals. BMC Health Services Research 25(1), 855. 10.1186/s12913-025-12983-4 40597294 PMC12219968

[r25] Kohrt BA, Gurung D, Singh R, Rai S, Neupane M, Turner EL, et al. (2025) Is there a mental health diagnostic crisis in primary care? Current research practices in global mental health cannot answer that question. Epidemiology and Psychiatric Sciences 34, e7. 10.1017/s2045796025000010 39880821 PMC11822449

[r26] Kohrt BA, Kunz RD, Baldwin JL, Koirala NR, Sharma v D and Nepal MK (2005) Somatization and comorbidity: A study of Jhum-Jhum and depression in rural Nepal. Ethos 33(1), 125–147. 10.1525/eth.2005.33.1.125

[r27] Kohrt BA, Ojagbemi A, Luitel NP, Bakolis I, Bello T, McCrone P, et al. (2025) An app-based WHO mental health guide for depression detection: A cluster randomized clinical trial. JAMA Network Open 8(5), e2512064. 10.1001/jamanetworkopen.2025.12064 40408108 PMC12102703

[r28] Levesque JF, Harris MF and Russell G (2013) Patient-centred access to health care: Conceptualising access at the interface of health systems and populations. International Journal for Equity in Health 12(1), 18. 10.1186/1475-9276-12-18 23496984 PMC3610159

[r29] Luitel NP, Breuer E, Adhikari A, Kohrt BA, Lund C, Komproe IH and Jordans MJD (2020) Process evaluation of a district mental healthcare plan in Nepal: A mixed-methods case study. BJPsych Open 6(4), e77. 10.1192/bjo.2020.60 32718381 PMC7443901

[r30] Luitel NP, Garman EC, Jordans MJD and Lund C (2019) Change in treatment coverage and barriers to mental health care among adults with depression and alcohol use disorder: A repeat cross sectional community survey in Nepal. BMC Public Health 19(1), 1350. 10.1186/s12889-019-7663-7 31640647 PMC6806507

[r31] Luitel NP, Jordans MJ, Adhikari A, Upadhaya N, Hanlon C, Lund C and Komproe IH (2015) Mental health care in Nepal: Current situation and challenges for development of a district mental health care plan. Conflict and Health 9(1), 3. 10.1186/s13031-014-0030-5 25694792 PMC4331482

[r32] Luitel NP, Jordans MJD, Kohrt BA, Rathod SD and Komproe IH (2017) Treatment gap and barriers for mental health care: A cross-sectional community survey in Nepal. PLoS One 12(8), e0183223. 10.1371/journal.pone.0183223 28817734 PMC5560728

[r33] Luitel NP, Jordans MJD, Subba P and Komproe IH (2020) Perception of service users and their caregivers on primary care-based mental health services: A qualitative study in Nepal. BMC Family Practice 21(1), 202. 10.1186/s12875-020-01266-y 32988367 PMC7523041

[r34] Luitel NP, Lamichhane B, Koirala P, Sainju P, Ghimire A, Gautam K, et al. (2025) Treatment of depression by traditional faith healers in Nepal: A qualitative study. SSM - Mental Health 7, 100425. 10.1016/j.ssmmh.2025.100425 40519523 PMC12163705

[r35] Luitel NP, Pudasaini K, Pokhrel P, Lamichhane B, Gautam K, Adhikari S, et al. (2023) Development and functioning of the mobile app-based mh-GAP intervention guide in detection and treatment of people with mental health conditions in primary healthcare settings in Nepal. Global Mental Health 10, e90. 10.1017/gmh.2023.69 38161752 PMC10755379

[r36] Lynn B, Dahal DR and Govindasamy P (2008) Caste, Ethnic and Regional Identity in Nepal: Further Analysis of the 2006 Nepal Demographic and Health Survey. Calverton, MD: Macro International Inc.

[r37] Mahat A, Citrin D and Bista H (2018) NGOs, partnerships, and public-private discontent in Nepal’s health care sector. Medicine Anthropology Theory 5(2), 100–126. 10.17157/mat.5.2.529

[r38] MoHP (2020) National Mental Health Strategy and Action Plan 2020. Kathmandu: Government of Nepal Ministry of Health and Population

[r39] National Statistics Office (2023) National Population and Housing Census 2021. Kathmandu: Government of Nepal, Office of the Prime Minister and Council of Ministers. https://censusnepal.cbs.gov.np/results/literacy (accessed 30 April 2023).

[r40] Patel V, Saxena S, Lund C, Thornicroft G, Baingana F, Bolton P, et al. (2018) The lancet commission on global mental health and sustainable development. Lancet 392(10157), 1553–1598. 10.1016/s0140-6736(18)31612-x 30314863

[r41] Paudel S, Chalise A, Khatri D, Poudel S and Khanal A (2025) Nepal’s mental health system from public health perspective: A thematic synthesis based on health system building blocks. Lancet Regional Health Southeast Asia 36, 100588. 10.1016/j.lansea.2025.100588 40370428 PMC12076720

[r42] Penchansky R and Thomas JW (1981) The concept of access. Medical Care 19(2), 127–140. 10.1097/00005650-198102000-00001 7206846

[r43] Penninx BW, Nolen WA, Lamers F, Zitman FG, Smit JH, Spinhoven P, et al. (2011) Two-year course of depressive and anxiety disorders: Results from the Netherlands study of depression and anxiety (NESDA). Journal of Affective Disorders 133(1–2), 76–85. 10.1016/j.jad.2011.03.027 21496929

[r44] Petersen I, van Rensburg A, Kigozi F, Semrau M, Hanlon C, Abdulmalik J, et al. (2019) Scaling up integrated primary mental health in six low- and middle-income countries: Obstacles, synergies and implications for systems reform. BJPsych Open 5(5), e69. 10.1192/bjo.2019.7 31530322 PMC6688466

[r45] Rai Y, Gurung D and Gautam K (2021) Insight and challenges: Mental health services in Nepal. BJPsych International 18(2), E5. 10.1192/bji.2020.58 34287402 PMC8274424

[r46] Raviola G, Naslund JA, Smith SL and Patel V (2019) Innovative models in mental health delivery systems: Task sharing care with non-specialist providers to close the mental health treatment gap. Current Psychiatry Reports 21(6), 44. 10.1007/s11920-019-1028-x 31041554

[r47] Shakya DR (2021) Observation of telepsychiatry service in a teaching hospital of eastern Nepal during COVID-19 pandemic. Insights Depress Anxiety 5(1), 025–028. 10.29328/journal.ida.1001027

[r48] Singh R, Chhetri K, Khanal P, Maharjan S, Garman E, Jordans MJD, et al. (2025) Assessment and management of suicidality in a mental health survey among poverty affected adolescents in Nepal. BMC Public Health 25(1), 3612. 10.1186/s12889-025-24943-y 41146168 PMC12560324

[r49] Subba P, Luitel NP, Kohrt BA and Jordans MJD (2017) Improving detection of mental health problems in community settings in Nepal: Development and pilot testing of the community informant detection tool. Conflict and Health 11(1), 28. 10.1186/s13031-017-0132-y 29181088 PMC5694900

[r50] Troup J, Fuhr DC, Woodward A, Sondorp E and Roberts B (2021) Barriers and facilitators for scaling up mental health and psychosocial support interventions in low- and middle-income countries for populations affected by humanitarian crises: A systematic review. International Journal of Mental Health Systems 15(1), 5. 10.1186/s13033-020-00431-1 33413526 PMC7792016

[r51] Upadhaya N, Regmi U, Gurung D, Luitel NP, Petersen I, Jordans MJD and Komproe IH (2020) Mental health and psychosocial support services in primary health care in Nepal: Perceived facilitating factors, barriers and strategies for improvement. BMC Psychiatry 20(1), 64. 10.1186/s12888-020-2476-x 32054462 PMC7020582

[r52] van den Broek M, Gandhi Y, Sureshkumar DS, Prina M, Bhatia U, Patel V, et al. (2023) Interventions to increase help-seeking for mental health care in low- and middle-income countries: A systematic review. PLOS Global Public Health 3(9), e0002302. 10.1371/journal.pgph.0002302 37703225 PMC10499262

[r53] Wang PS, Angermeyer M, Borges G, Bruffaerts R, Tat Chiu W, et al. (2007) Delay and failure in treatment seeking after first onset of mental disorders in the World Health Organization’s World Mental Health Survey Initiative. World Psychiatry 6(3), 177–185. 18188443 PMC2174579

[r54] WHO (2010) mhGAP Intervention Guide for Mental, Neurological and Substance Use Disorders in Non-specialized Health Settings: Mental Health Gap Action Programme (mhGAP) – Version 1.0. Geneva: WHO. 23741783

[r55] WHO (2016) mhGAP Intervention Guide for Mental, Neurological and Substance Use Disorders in Non-specialized Health Settings: Mental Health Gap Action Programme (mhGAP) – Version 2.0. Geneva: World Health Organization. 27786430

[r60] WHO (2023) Mental Health Gap Action Programme (mhGAP) Guideline for Mental, Neurological and Substance use Disorders. Geneva: World Health Organization. https://www.who.int/publications/i/item/B09329. 38117918

