# Supplementary material for: Breaking down barriers: A qualitative study of demand- and supply-side barriers to depression care in Nepal
Source: Glob Ment Health (Camb). 2026 May 28;13:e121. doi: 10.1017/gmh.2026.10236 (PMC13279957; doi:10.1017/gmh.2026.10236)
Supplement: Peer review history

# Reviewer report: Review: Breaking down barriers: A qualitative study of demand- and supply-side barriers to depression care in Nepal — R0/PR2

**DOI:** 10.1017/gmh.2026.10236.pr2

The study builds on the gaps of earlier studies that did not consider supply side and demand side barriers utilizing a hybrid approach based on access framework by Levesque and colleagues (health system characteristics and individual abilities). The manuscript is well drafted and bears no major revision areas. However, minor modifications may be required for on the followings:

1. In Methods section Settings subsection, It would be better to provide references or further information to support statements from Line 9-16. These can help an unknown understand the context of Nepal and mental health service in Nepal clearly. What diverse population and healthcare contexts do the authors mean?

2. In study design, the statement feels incomplete.

3.Non-maleficence are generally missed or overlooked by many researchers in their studies. In Methods section Interview process subsection, can the authors mention what precautions were considered by the team to not cause non-maleficence as “Individuals receiving care were identified through PHC workers and interviewed either at home or in a confidential clinical setting, based on preference” in the study?

4. In Data Management and Analysis subsection, whether any objective procedure could also be used to resolve discrepancies?

5. In Line 16, Page 15 of the manuscript, either additionally or similarly needs to be removed.

# Reviewer report: Review: Breaking down barriers: A qualitative study of demand- and supply-side barriers to depression care in Nepal — R0/PR3

**DOI:** 10.1017/gmh.2026.10236.pr3

Thank you for asking me to review this interesting article that is reporting on barriers to depression care in Nepal. I enjoyed reading the paper, and have a few minor suggestions below:

1. Authors used Levesque’s framework to help interpret the results. I find this framework a bit difficult to conceptualise from a demand and supply side. I also didn’t like the way the framework listed implementation outcomes for their categories of determinants. Do you credit, you described nicely how the different barriers fit into this framework. If you are going to use a framework in the future, I would consider using a more recent framework that categorises context into domains such as the external setting, the internal setting, the intervention, the implementation of the intervention, and the users. The Context and Implementation of Complex Interventions framework has particular relevance in LMIC settings such as Nepal. For instance, authors for this paper describe the external context in great detail in the appendix that describes how sociocultural, socioeconomic determinants can influence the implementation of an intervention. Although you are not implementing an intervention, it is still highly relevant.

2. Please keep in mind that there are many social scientists out there who do not appreciate frameworks. ALthough I have used frameworks in the past, I do now recognise their limitations. Context is extremely complex, and the categorisation of determinants undermines this. I would consider putting a few sentences in the limitations about this in anticipation of this criticism.

3. On page for some papers were referenced that were pretty outdated. I would consider using more up to date articles. For example, Wang et al 2007 was used to describe waiting times.

4. Table 1 on page five is a bit difficult to interpret. You have presented the percentages in rows, these should be column percentages presented for each category. For example, in Kailalo display the percentage for different categories of participant type.

5. There are a few copy editing mistakes in the paper. For example on page 9, you present [hospital name].

6. I would also consider reporting on the enablers, and not just the barriers (if you have this information). Very often this information is overlooked which is problematic. Importantly, enablers help you better understand what is working well within a health system, and how it can be leveraged to help with improving things.

Well done on an interesting and important paper.

# Editor report: Recommendation: Breaking down barriers: A qualitative study of demand- and supply-side barriers to depression care in Nepal — R0/PR4

**DOI:** 10.1017/gmh.2026.10236.pr4

**Affiliations:** Heilbrunn Department of Population and Family Health, Columbia University, United States

Dear Dr. Luitel, 

The reviewers find the paper interesting and have suggested minor revisions. I invite you to submit after addressing these concerns. Thank you.

# Editor report: Decision: Breaking down barriers: A qualitative study of demand- and supply-side barriers to depression care in Nepal — R0/PR5

**DOI:** 10.1017/gmh.2026.10236.pr5

**Affiliations:** London School of Hygiene & Tropical Medicine, United Kingdom of Great Britain and Northern Ireland

No accompanying comment.

# Editor report: Recommendation: Breaking down barriers: A qualitative study of demand- and supply-side barriers to depression care in Nepal — R1/PR7

**DOI:** 10.1017/gmh.2026.10236.pr7

**Affiliations:** Heilbrunn Department of Population and Family Health, Columbia University, United States

Thank you for addressing the comments to the reviewers thoroughly.

# Editor report: Decision: Breaking down barriers: A qualitative study of demand- and supply-side barriers to depression care in Nepal — R1/PR8

**DOI:** 10.1017/gmh.2026.10236.pr8

**Affiliations:** London School of Hygiene & Tropical Medicine, United Kingdom of Great Britain and Northern Ireland

No accompanying comment.
